# Utilization of the less-invasive stabilization system internal fixator for open fractures of the proximal tibia: A multi-center evaluation

**DOI:** 10.4103/0019-5413.43390

**Published:** 2008

**Authors:** James P Stannard, Christopher G Finkemeier, Jackson Lee, Philip J Kregor

**Affiliations:** University of Alabama at Birmingham, Birmingham, Alabama, USA; 1 Sutter Roseville Medical Center, Roseville, California, USA; 2 University of Southern California, Los Angeles, California, USA; 3 Vanderbilt University, Nashville, Tennessee, USA

**Keywords:** Less Invasive Stabilization System, Open fractures, proximal tibia

## Abstract

**Background:**

Locked plating has become popular and has clear biomechanical advantages when compared with conventional plating. When combined with minimally invasive surgical techniques, locked plating may cause substantially less iatrogenic tissue damage when compared with conventional plating. These characteristics may make locked plating an attractive option for treating open fractures of the tibial plateau and proximal tibia for which coverage over the plate can be obtained. The purpose of this study was to evaluate the use of the Less-Invasive Stabilization System (LISS) for high-energy open fractures involving either the tibial plateau or proximal tibia.

**Materials and Methods:**

This study is a retrospective evaluation of a consecutive multicenter series of 52 consecutive patients operated by seven surgeons, who used LISS plating in open proximal tibia or tibial plateau fractures seen at one of four Level I Trauma Centers. All patients were treated using a locked plating system that was implanted using minimally invasive submuscular surgical techniques. The primary outcome measure was the incidence of deep and superficial infection.

**Results:**

Fifty-two patients with open fractures have been evaluated, with a mean follow-up of 16.8 (12–36) months. Three patients (5.8%) developed deep infections. Two patients (6.3%) with tibial plateau and one (4.3%) of patients with a tibial shaft fracture developed deep infections. Fifteen patients required flap coverage of their open wounds. The incidence of deep infection as per Gustilo and Anderson classification was Type I and II – 0 (0%); Type IIIA – 2 (7.7%); Type IIIB – 1 (7.1%); and Type IIIC – 0 (0%).

**Conclusions:**

Biomechanically, the LISS functions as an “internal-external fixator” rather than a plate. Traditional plate osteosynthesis has yielded rates of infection between 18% and 35%. Our data indicate that locked plating using minimally invasive techniques yield deep infections rates that are no worse than published series using intramedullary nails or external fixators. Technical difficulties that can be encountered with the LISS system revolve primarily around obtaining and maintaining reduction while performing a minimally invasive procedure. Additional difficulties can include “cold welding” of screws to the plate and malposition of the plate leading to failure in the diaphysis. High-energy open fractures involving the tibia shaft or plateau remain high-risk injuries, but LISS is an acceptable alternative for treatment of these fractures.

## INTRODUCTION

Open fractures involving the tibial plateau or tibial shaft are severe injuries often associated with complications including skin breakdown and superficial or deep infection.^1^–^14^ A remarkable evolution of recommended treatment options has occurred over the past 50 years, as techniques of surgical stabilization of fractures and implants available have developed. Clearly, each fracture requires individualized decision-making, and there are a large number of variables that can dictate what the ideal implant should be for an individual fracture. However, there is a general consensus that either intramedullary nails^5^,^10^,^15^,^16^ or external fixators^1^–^3^,^6^,^12^–^14^ have a lower risk of infection than conventional compression plating techniques.^1^,^3^,^4^,^9^,^11^

The Less Invasive Stabilization System (LISS) (Synthes USA, Paoli, Pennsylvania) is a new implant system that has been developed by the AO group. It was initially developed for supracondylar femur fractures and has now been expanded to include a set of implants for tibial plateau and some tibial shaft fractures. Although its external appearance is very similar to a conventional plate, it functions biomechanically more like an external fixator.^17^ Indeed, it has been referred to as an internal-external fixator because it is implanted beneath the skin yet it functions like an external fixator.^17^

The purpose of this study is to evaluate the use of the LISS plates and screws with open fractures involving the tibial plateau or proximal tibia. Our hypothesis is that the LISS plates provide adequate stabilization for either tibia plateau or proximal tibia shaft fractures with no greater risk of infection than external fixation or intramedullary nailing.

## MATERIALS AND METHODS

Between November 1998 and July 2001, 52 patients with open fractures of the tibial plateau, shaft, or a combination of both were treated with LISS implants at four different Level I Trauma Centers. This study is a retrospective evaluation of prospectively collected data from the four centers. The fractures were classified using the AO/OTA (Orthopedic Trauma Association) classification, and open fractures classified were classified according to the method of Gustilo and coworkers.^18^ Implant selection was entirely at the discretion of the attending surgeon. Fracture “personality” or characteristics played a big role in the decision to use locked plating. Bicondylar fractures of the tibial plateau were treated with locked plating at all of the involved centers. Open unicondylar fractures were also treated with locked plating by the authors, taking advantage of the minimally invasive technique and the lack of compression of the periosteal blood supply by the plate. Open tibial shaft fractures that had proximal or distal extension that made intramedullary nailing difficult were also included in this treatment algorithm. The time period of this study reflects the initial use of locked plating. This study represents only the patients of seven surgeons at the four centers who were using locked plating, and not the entire trauma load seen by their respective trauma centers. Infection was diagnosed based on clinical signs of infection including erythema and drainage, as well as the systemic signs of infection. All deep infections were confirmed by culture.

The protocol of all four centers involved either immediate stabilization with the LISS implant or one or more irrigation and debridements, followed by definitive stabilization on a delayed basis. Patients treated with the delayed protocol were temporarily splinted with a knee immobilizer if there was no shortening of the fracture and the leg was relatively stable. If the fracture did not meet those criteria, a spanning external fixator was applied until definitive stabilization was achieved with the LISS implant. The decision regarding the timing of placing the LISS implant was made by the senior surgeon involved with the case. All flaps were done in conjunction with placing the LISS implant for definitive stabilization of the fracture. This series includes all open LISS cases involving the tibia at all four Level I Trauma Centers, including our initial experience or learning curve with the implant.

The LISS utilizes precontoured anatomically shaped plates made of a titanium alloy. The screws combine a drill bit tip and a tap and are designed to be implanted percutaneously using a power drill. The screw heads are threaded and lock into the screw holes when fully implanted, forming a fixed angle construct analogous to a blade plate. Screws are available in a limited range of lengths and are designed to be unicortical. The combination of unicortical screws that lock into the implant yields no compression at the interface between the plate and the bone. The net result is that the plate does not compress the periosteal vessels when implanted. In this respect, the plate functions more like an “internal-external fixator” than like a conventional plate.^17^ LISS technique for open fractures is identical to the technique used for closed fractures, with the exception of the irrigation and debridement prior to the implantation of the LISS. All LISS fixators were placed on the lateral side of the tibia, and pinned proximally and distally. Length was selected to obtain a minimum of three good screws distal to the fracture, with a preference for at least four screws if the fracture allowed. All diaphyseal screws were placed using the irrigation system to cool while drilling. Great care was taken to employ gentle soft tissue handling and minimize incisions and soft tissue damage.

Patients in this study were treated at four different trauma centers, each with slightly different protocols. The majority of the patients were from one center, the University of Alabama at Birmingham. Our protocol involved irrigation and debridement within eight hours of admission, with definitive stabilization with the LISS plate at that time if the soft tissue injury and wound allowed. If there was too much of soft tissue damage or contamination, patients were taken back to the operating room within 48–72 h for a repeat irrigation and debridement. LISS stabilization was accomplished at that point if the soft tissues allowed. If not, the process of irrigation and debridement and soft tissue assessment was repeated every 48–72 h until the LISS was applied. Antibiotic prophylaxis was provided for the first 72 h following injury, and for every 24-h period following surgeries. A first-generation cephalosporin was used for Type I and II fractures. Patients with Type III fractures had gentamicin added to the cephalosporin, or single antibiotic treatment with a broad-spectrum antibiotic.

## RESULTS

Fifty-four patients with open fractures were treated with locked plating using minimally invasive techniques at four Level I Trauma Centers. Patients with 52 open fractures of the tibial shaft or plateau with a minimum 12-month follow-up were included in this study. There were 41 male and 11 female patients enrolled. The mean age of our patients was 38 years, with a range of 17–76 years. The mean follow-up was 16.8 months, with a range of 12–36 months. The mechanisms were primarily high-energy injuries with 25 patients involved in motor vehicle accidents; 10 in pedestrian versus motor vehicle accidents; nine ballistic injuries; three assaults; two falls from a substantial height; two crush injuries; and one patient involved in an airplane crash.

**Figure 1:**
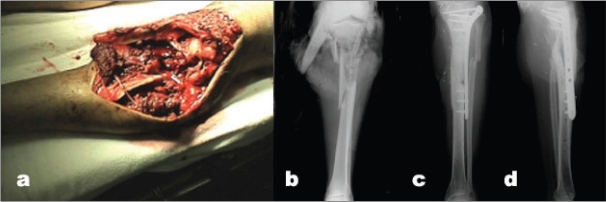
(a) Clinical photograph shows IIIC tibial plateau and shaft fracture. (b) Anteroposterior X-ray of the leg shows type IIIC tibial plateau and shaft fracture. (c,d) Anteroposterior and lateral radiograph at 6-year follow-up shows fracture union.

Thirty-two fractures involved the tibial plateau and 24 involved the diaphyseal region of the bone. (Diaphyseal fractures with extension toward either the knee or ankle were a common pattern treated with locked plating in this study). Additionally, diaphyseal fractures that were already exposed because of the open wound were considered for locked plating. The third category of diaphyseal fracture that we evaluated for locked plating involved patients who were active in jumping or kneeling activities at either work or recreational activities. Knee pain associated with intramedullary nailing was discussed with the patient as we determined the ideal treatment plan. Four patients sustained fractures that involved both the plateau and the diaphysis. The AO/OTA classification of our fractures is described in Table 1. The open fracture classification included: Type I in three patients; Type II in seven patients; and Type III in 42 patients. When the type III fractures are subclassified,^18^ we had 26 Type IIIA fractures, 14 Type IIIB fractures, and two Type IIIC fractures. Both Type IIIC fractures had irrigation and debridement followed by vascular surgery performed to reestablish arterial flow. The LISS implant was then placed to stabilize the fracture. Both of these fractures healed without complications [Figure 1]. The time from injury to definitive stabilization of the fracture with a LISS implant was a mean of 2.9 (0-17) days. Thirteen patients had their fracture stabilized with a LISS implant on the day of injury, and an additional 12 had LISS stabilization within 24 h. Fifteen patients required flaps to obtain soft tissue coverage of their injury, including 13 rotational flaps and two free flaps. The rotational flaps included sural, gastrocnemius, and soleus flaps.

**Table 1:** AO/OTA fracture classification breakdown

AO/OTA classification	Number of patients
41A	4
41B	2
41C	26
42A	3
42B	5
42C	16

Three patients (5.8%) in our series developed deep infections or osteomyelitis requiring multiple surgical irrigation and debridement procedures. There were two deep infections involving the tibial plateau, and one involving the tibial shaft. The incidence of deep infection when broken down by anatomic region was 6.3% for tibial plateau and 4.3% for tibial shaft fractures. When evaluated in terms of open fracture classification, there were two deep infections in patients with IIIA fractures and one in a patient with a IIIB fracture. One patient developed chronic osteomyelitis and a nonunion that was not responsive to repetitive irrigation and debridements with the use of antibiotic beads. He also had several courses of six or more weeks of antibiotic therapy based on the results of the bacterial sensitivity testing. This patient was definitively treated with a below the knee amputation and represents the only patient in this series that received an amputation. An additional three patients developed superficial infections. Two of these were treated only with antibiotics, while one underwent a single surgical procedure to evacuate and irrigate a hematoma. As mentioned above, one patient with an infection developed a nonunion and two additional patients developed nonunions that required additional bone grafting to obtain union. There was no evidence of infection in these later two patients, including negative cultures at the time of surgery. There were no patients in this study who had implant failure or loss of fracture reduction that was achieved in the operating room.

## DISCUSSION

Open fractures of the tibia and tibial plateau are severe osseous and soft tissue injuries associated with a high rate of complications, notably infection and nonunion.^1^–^14^ One major reason that the tibia is associated with these risks is the limited soft tissue coverage of the bone and associated limited blood supply.^19^ The lack of access to the open fracture site, lack of stability, and subsequent damage to the soft tissues have led to a trend of surgical stabilization of open tibia shaft and plateau fractures, rather than definitive management with casting and/or traction.^8^,^20^ Compression plate fixation using AO techniques has been advocated,^8^ but a number of surgeons have reported a high incidence of infection.^1^,^3^,^4^,^7^,^9^,^11^ The rate of deep infections and/or osteomyelitis has ranged from 11 to 80%,^1^–^4^,^7^–^9^,^11^ with most authors reporting rates of 18% or more.^1^–^3^,^7^,^9^,^11^ The infection rates reported with plating are high for both tibial shaft fractures (11–40%)^1^–^4^,^8^,^9^ and tibial plateau fractures (33–80%).^7^,^11^ One problem with compression plates is that they devitalize bone under the plate. This occurs both as a result of direct compression of the periosteal vessels,^19^,^21^–^23^ as well as a disturbance of the flow of blood between the endosteal and periosteal systems,^19^ causing significant devascularization between both.^19^,^21^–^23^ This devitalization and necrosis of bone as a result of compression plating may increase the risk of infection in the face of open fractures.

High-energy tibial plateau fractures are severe injuries frequently associated with a high incidence of infection and soft tissue breakdown. As a result of the problems associated with plating described above, a number of recent studies have been reported utilizing small wire or hybrid external fixators. Deep infection and osteomyelitis remain a significant problem, with rates of 7–13%.^6^,^12^–^14^ Kumar and Whittle reported only a 7% incidence of deep infection, yet had amputations in three of those four patients.^6^ Superficial or pin tract infections requiring only antibiotic treatment are reported in 25–100% of patients.^6^,^13^,^24^ Although the incidence of infection has clearly been better than that reported with plating, a number of other complications have been reported, including malunion,^6^,^14^,^24^ motion problems,^6^,^14^,^24^,^25^ and septic arthritis.^13^,^25^

Open diaphyseal fractures of the tibia are also associated with a high incidence of infection, osteomyelitis, and nonunion. As a result, two primary surgical treatment strategies have evolved: external fixation^1^–^3^,^5^,^16^ and intramedullary nailing.^10^,^15^,^16^ The incidence of infection reported with external fixation of the tibial shaft varies from 3–14%.^1^,^3^,^5^,^16^ Additional problems associated with external fixation have included pin tract infections, malunions, and nonunions.^1^,^3^,^5^,^10^,^16^ Recently, there has been a trend toward the use of either reamed or unreamed intramedullary nails as a treatment for open fractures of the tibia.^10^,^15^,^16^ Infection rates reported for all open fractures have varied from 3 to 8%, with a rate of 12% for IIIA and 25% for IIIB reported by Whittle *et al*.^10^ Advantages of intramedullary nailing includes eliminating the problem with pin tract infections and decreasing the risk of malunion.

The LISS implants represent another option in the treatment of open fractures of the tibial plateau and shaft. The combination of minimal soft tissue dissection, small surgical approaches, and plates that do not compress the bone yield an implant that would be expected to yield a lower rate of infection than conventional plates. The unicortical screws have threaded heads that lock into the plate, creating a fixed angle implant.^17^ The implant does not depend on compression between the plate and the bone, which is different from other plate and screw constructs. This spares both the endosteal and periosteal blood supplies to the tibia.^19^,^21^–^23^,^26^–^28^ While the LISS implants might be expected to yield a lower rate of infection in open tibia shaft and plateau fractures, there have been no published series to date specifically evaluating the use of the system in open fractures.

Our data combined from four separate trauma centers and seven different attending surgeons indicates that the use of the LISS implants in open fractures results in a rate of infection that is not higher than the use of other contemporary methods of stabilization. Our overall rate of infection of 5.8% compares favorably with most of the references cited above. The incidence of infection of 6.3% with high-energy bicondylar tibial plateau fractures is remarkably better than some series using plate osteosynthesis^7^,^11^ and at least as good as most contemporary series using small wire or hybrid external fixators.^6^,^7^,^12^–^14^ Similarly, our 4.3% rate of infection for open tibial shaft fracture is at least as good as the contemporary studies using locked nailing.^10^,^29^,^30^ Similarly, our 5.8% incidence of nonunion compares well with many other studies.^1^,^2^,^10^

The authors recognize that in some respects combining tibia shaft and tibial plateau fractures is like combining apples and oranges. However, in terms of infection, both injuries have a high incidence of infection. Additionally, some fractures span both regions of the bone as demonstrated in four of our patients. We do not intend to leave the reader with the impression that we think that LISS implants have now replaced other methods as the preferred treatment of open fractures of the tibial diaphysis and plateau. We believe that LISS may be extremely beneficial for bicondylar tibial plateau fractures, proximal tibia fractures that do not involve the joint, and combined shaft/plateau fractures [Figure 1]. We continue to utilize other methods of stabilization for most open tibia diaphyseal fractures. However, our results indicate that LISS is an acceptable implant for use in open fractures of the tibia, as we expected based on the biomechanics of the device. The LISS provides another tool for orthopedic trauma surgeons to utilize in stabilizing these challenging open fractures.
